# A male-produced aggregation-sex pheromone of the beetle *Arhopalus rusticus* (Coleoptera: Cerambycidae, Spondylinae) may be useful in managing this invasive species

**DOI:** 10.1038/s41598-019-56094-7

**Published:** 2019-12-20

**Authors:** Alenka Žunič-Kosi, Nataša Stritih-Peljhan, Yunfan Zou, J. Steven McElfresh, Jocelyn G. Millar

**Affiliations:** 1 National Institute of Biology, Department of Organisms and Ecosystem Research, Ljubljana, Slovenia; 2 University of California, Department of Entomology, Riverside, California, United States of America

**Keywords:** Invasive species, Invasive species

## Abstract

The longhorned beetle *Arhopalus rusticus* (Coleoptera: Cerambycidae, Spondylinae) is a common species in conifer forests of the Northern Hemisphere, but with global trade, it has invaded and become established in New Zealand, Australia, and South America. *Arhopalus rusticus* is a suspected vector of the phytopathogenic nematode, *Bursaphelenchus xylophilus*, the causative agent of pine wilt disease, which is a major threat to pine forests worldwide. Here, we report the identification of a volatile, male-produced aggregation-sex pheromone for this species. Headspace odours from males contained a major male-specific compound, identified as (2 *S*, 5*E*)-6,10-dimethyl-5,9-undecadien-2-ol (common name (*S*)-fuscumol), and a minor component (*E*)-6,10-dimethyl-5,9-undecadien-2-one (geranylacetone). Both compounds are known pheromone components for species in the same subfamily. In field trials in its native range in Slovenia, (*S*)-fuscumol was significantly more attractive to beetles of both sexes, than racemic fuscumol and a blend of host plant volatiles commonly used as an attractant for this species. Fuscumol-baited traps also caught significant numbers of another spondylidine species, *Spondylis buprestoides* (L.), and a rare click beetle, *Stenagostus rufus* (De Geer). The pheromone can be exploited as a cost-effective and environmentally safe tool for detection and monitoring of this invasive species at ports of entry, and for monitoring the beetle’s distribution and population trends in both endemic and invasive populations.

## Introduction

Insects are among the most common groups of invaders worldwide^1^. Species from the order Coleoptera are the most frequently intercepted exotic insects^2–4^, being transported on or in commodities such as logs, lumber, wooden packing material, live plants, seeds, and food items. Non-indigenous beetles have the potential, alone or in combination with pathogens, to cause massive disturbance to forest ecosystems, including their biophysical and biochemical processes, community composition, and biodiversity^5^. In addition, invasive beetles can also greatly influence the economics of natural and plantation forests, timber production, recreational forests, and ornamental trees planted for shade and aesthetic value^6,7^. During the past decades, increasing numbers of forest insect incursions and establishments have been recorded all around the world, especially for bark and wood-boring beetles from the families Buprestidae and Scolytidae^3^. Recently, several wood-boring longhorned beetles (Cerambycidae) also have successfully invaded new countries and new continents^8,9^. As such, they are receiving increasing regulatory and public attention (e.g.^10–12^), with intensifying efforts to detect, monitor, and eradicate invasive species. However, there are currently no reliable methods for early detection of exotic cerambycid species, whose larvae are hidden deep in the wood of imported wooden products. Better surveillance methods are essential for early detection of larvae, or of adult beetles emerging from infested shipments, so that appropriate containment and eradication methods can be initiated.

The longhorn beetle *Arhopalus rusticus* (Linnaeus) (Coleoptera: Cerambycidae, Spondylidinae) is common in coniferous forests in Europe, North Africa, North America, Siberia, Korea, Tajikistan, Mongolia, Japan, North China^13–16^. In some earlier literature, this species was referred to as *A. tristis* but the two species have now been synonymized as *A. rusticus*^14,17^. Also, there are several recognized subspecies of *A. rusticus*, such as the North American *A. rusticus montanus*, *A. rusticus nubilus*, *A. rusticus hesperus*, and *A. rusticus obsoletus*, and the European and North Asian subspecies *A. rusticus rusticus*, which was the subject of this study. *A*. *rusticus* is commonly associated with pine (*Pinus*) forests but also develops in other coniferous trees, such as *Picea, Abies, Larix, Cupressus, Cryptomeria*, and *Juniperus*^18^.

Larvae develop subcortically in stressed, dying, or dead trees, fallen or standing trunks of large diameter, and in stumps or shallow roots^18^. As such, *A. rusticus* can cause tree decline, and by damaging structural timber, degrade the value of timber production^13,19^. Species in the genus *Arhopalus* have been recognized as significant pests of processed or fire- damaged *Pinus* trees worldwide^20,21^. In the 20^th^ century, several species in the genus *Arhopalus* including *A. rusticus*, *A. syriacus* (Reitter), and *A. ferus* (Mulsant), invaded Australia and New Zealand, causing damage in stressed pine trees and negatively affecting timber trade^10,14^. At the beginning of this century, *A. rusticus* also invaded Argentina^22,23^, and it has now dispersed throughout pine production areas in the province of Cordoba^22^.

Options available for management and eradication of alien species depend upon, among other factors, the biological attributes of specific species. In many insect species, mate finding and recognition are mediated by emission of pheromones^24,25^, and understanding this process is crucial for developing risk analyses, and for monitoring and management of invasive pest species^26^. In this context, the use of attractant pheromones and host plant volatiles have been shown to be among the most efficient means of detection, sampling, monitoring movement, and/or management of forest and timber exotic pests. Recent rapid progress in the chemical ecology of wood-boring cerambycids^8^ has demonstrated the potential for exploiting their pheromones for both surveillance and trapping of invasive pest species^27,28^.

Research to date has shown that the longhorned beetles rely heavily on chemicals present in their environment, and that the chemistry of their volatile pheromonal signals can exhibit considerable diversity^26^. However, we know little about the pheromone chemistry of cerambycids in the subfamily Spondylinae, with pheromones or possible pheromones (i.e., beetles are attracted but have not yet been shown to produce the compounds) identified from only a few species^26^.

Here, we collected headspace volatiles from *A. rusticus rusticus*, and identified the chemical components of the emitted odours. We found that males produce a blend of (*S*)-fuscumol as a sex-specific aggregation-sex pheromone, which was attractive to beetles of both sexes in field bioassays. The results of this study expand our knowledge on attractant pheromones for the subfamily Spondylinae, while also providing a crucial tool for improved monitoring and control of this cryptic but highly invasive forest insect pest.

## Results

**Figure 1:**
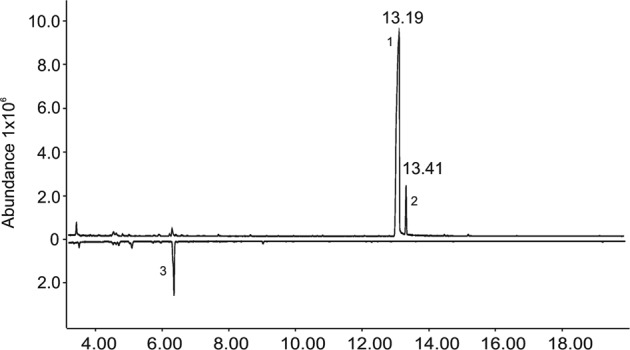
Representative total ion chromatograms (GC-MS) of the headspace volatiles collected from an *A. rusticus* male (top trace) and female (bottom, inverted trace). Two male-specific compounds from males are indicated by numbers 1 – (*S*)-fuscumol, 2 – geranylacetone. The compound indicated by number 3, identified as limonene, and other minor compounds in both traces were system contaminants.

**Figure 2:**
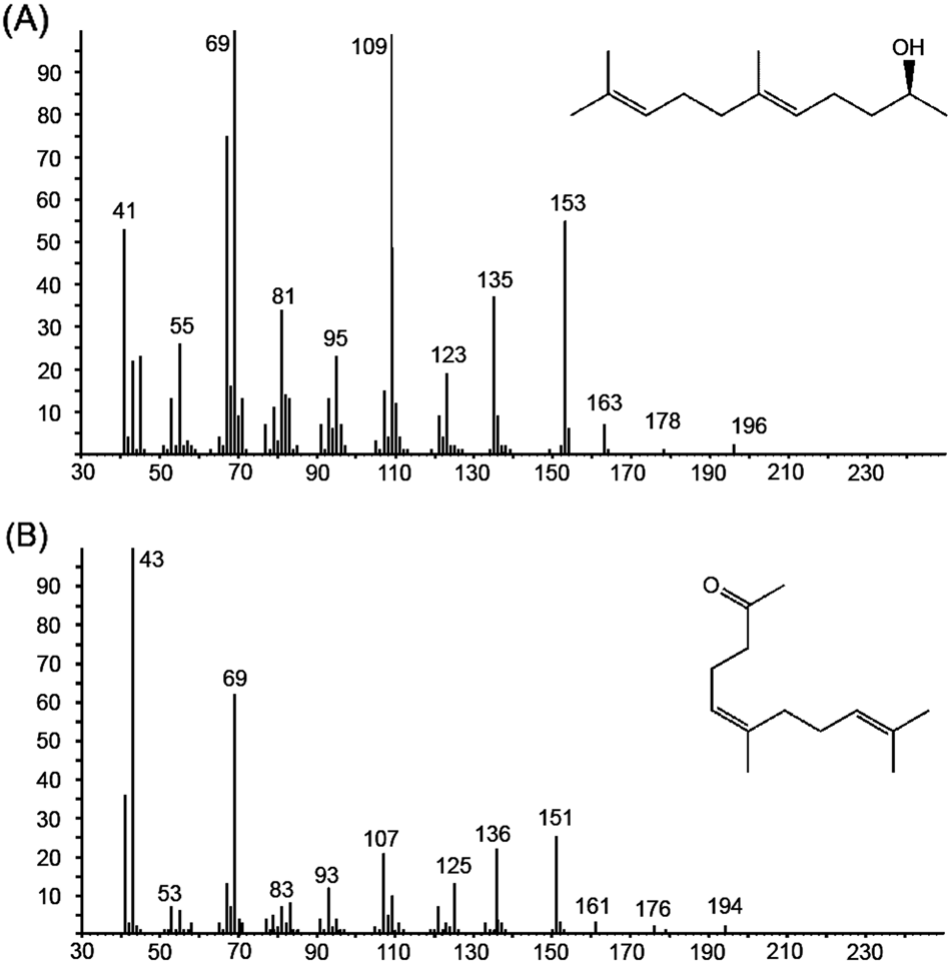
EI mass spectra of the two major compounds emitted by male *A. rusticus*; (**A**) (*S*)-fuscumol; (**B**) geranylacetone.

**Figure 3:**
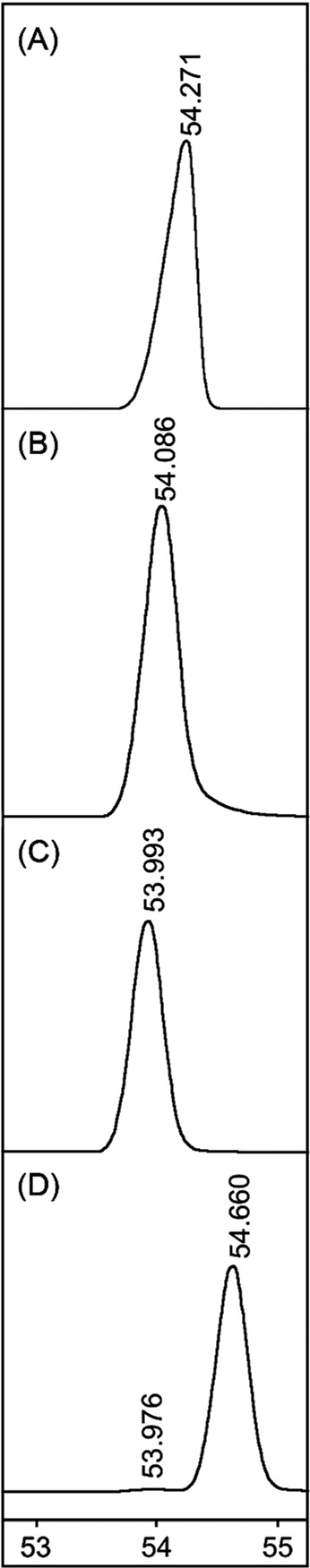
Analyses on a chiral stationary phase Cyclodex B GC column. (**A**) Insect extract after acetylation of fuscumol; (**B**) Acetylated insect extract coinjected with (*S*)*-*fuscumol acetate; (**C**) (*S*)-Fuscumol acetate; and (**D**) (*R*)-Fuscumol acetate.

While field testing a series of known cerambycid pheromones (AZK, unpub. data), we noticed that *A. rusticus* beetles were being caught specifically in traps baited with racemic fuscumol and its acetate, which was the first indication that the alcohol and/or its acetate might be pheromone compounds of this species. Live males and females were returned to the laboratory for collection of headspace volatiles. Three out of five extracts of headspace volatiles collected from males were dominated by two peaks, and no other insect-produced compounds were consistently present (Fig. 1). The major peak was identified as (*E*)-6,10-dimethyl-5,9-undecadien-2-ol (fuscumol) (diagnostic ions: *m*/*z* 196, 178, 109, 69) (Fig. 2A), and a second, minor peak as (*E*)-6,10-dimethyl-5,9-undecadien-2-one (geranylacetone, diagnostic ions: *m*/*z* 194, 69, 43) (Fig. 2B). The two compounds were released in about a 4:1 ratio (between 25% and 28% in the three extracts). Comparable extracts from females did not contain either of the two compounds, nor did they contain any female-specific compounds. The fact that only two compounds were consistently present in significant quantities in extracts of males suggested that these two components might be potential pheromone candidates. The absolute configuration of fuscumol in the extracts was determined with a chiral stationary phase Cyclodex B GC column. Because the alcohol enantiomers were not resolved, the insect-produced extract was acetylated (Fig. 3A). Reanalysis of the derivatized extract showed that the retention times of the acetylated insect-produced compound and (*S*)-fuscumol acetate matched exactly, whereas the retention time of the (*R*)-enantiomer was markedly different (Fig. 3), confirming that the insects produced exclusively the (*S*)-enantiomer of fuscumol.

**Figure 4:**
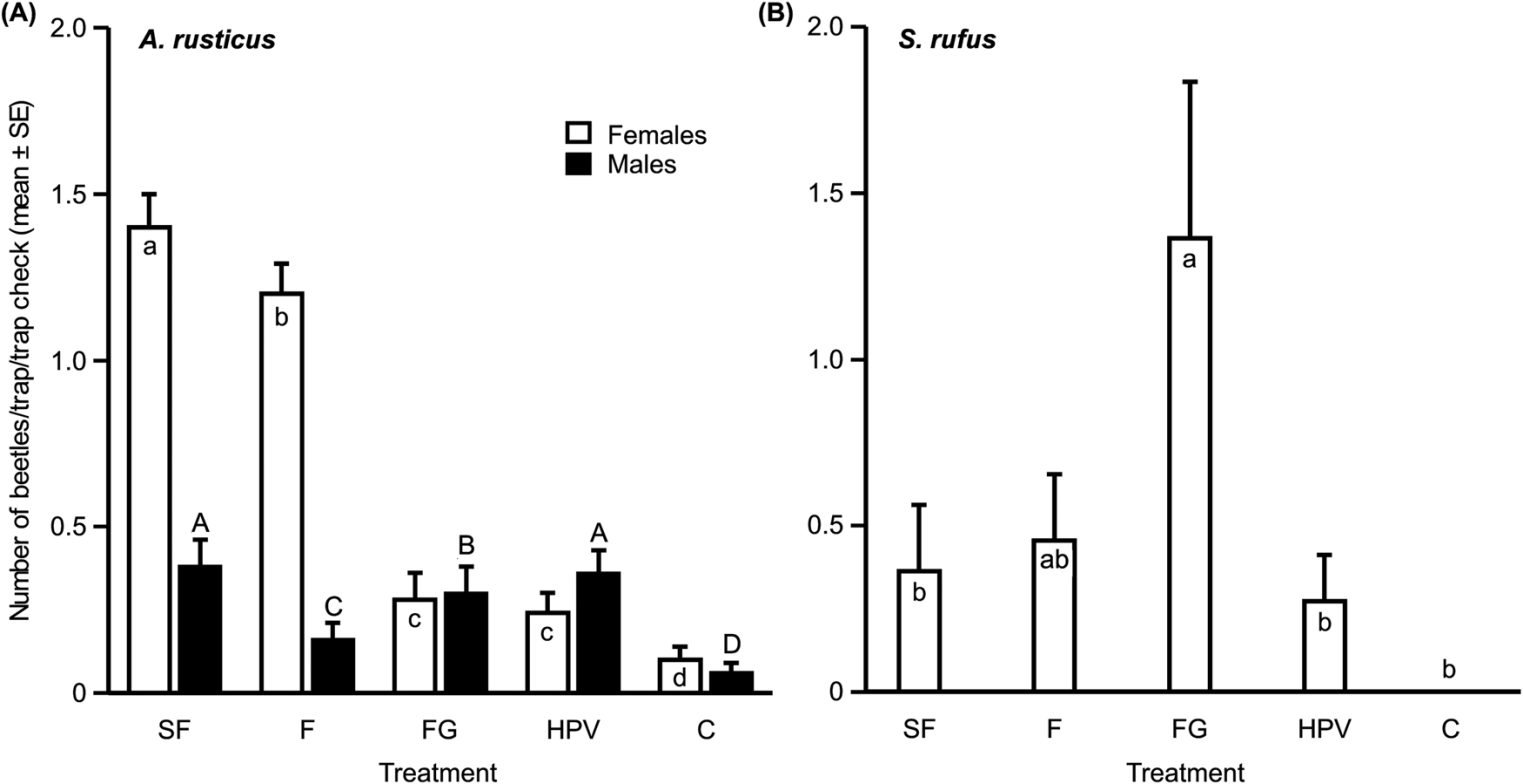
Mean ( ± standard error (SE)) numbers of (**A**) *A. rusticus* females (white bars; N_total_ = 161) and males (black bars; N_total_ = 63), and (**B**) *S. rufus* females (white bars; N_total_ = 27) captured/trap/trap check in traps baited with four test treatments (*S*)-Fuscumol (SF), racemic fuscumol (F), a blend of racemic fuscumol with geranylacetone (FG), host plant volatiles (HPV), and isopropanol as control (**C**) during a field bioassay in Slovenia in 2014. Number of replicates included in the analysis are 49, 36, and 12 for females, males, and *S. rufus*, respectively. Different letters indicate significant differences between treatments (Friedman’s test, Conover post hoc test, corrected by the Benjamini-Hochberg procedure, P < 0.05).

Field bioassays were carried out to test attraction to synthetic pheromone, and to compare attraction of *A. rusticus* females and males to racemic fuscumol, male-produced (*S*)-fuscumol, and the combination of fuscumol with geranylacetone. The host volatiles ethanol and α-pinene were included as an additional treatment, along with isopropanol-baited controls. In total, we captured 224 *A. rusticus* beetles (161 females and 63 males, sex ratio significantly different from 1:1, exact binominal test, P < 0.001). Lure treatments had a significant effect on the attraction of female (Friedman, χ^2^_(4)_ = 120.63, P < 0.001) as well as male beetles (Friedman, χ^2^_(4)_ = 18.38, P < 0.01) (Fig. 4A). (*S*)-fuscumol treatment attracted significantly more females (n = 70) than traps baited with either racemic fuscumol (n = 60), a blend of fuscumol and geranylacetone (n = 14), host plant volatiles (n = 12), or the control (n = 5) (Friedman, χ^2^_(4)_ = 120.63, Conover post hoc test with Benjamini-Hochberg correction, P < 0.01, for all comparisons) (Fig. 4A). (*S*)-fuscumol and the host plant volatiles were equally attractive to males (Friedman, χ^2^_(4)_ = 18.38, Conover post hoc test, with Benjamini-Hochberg correction, P = 0.69, catching 19 and 18 beetles, respectively) (Fig. 4A). Traps baited with racemic fuscumol captured significantly more females then a blend of fuscumol and geranylacetone, or host plant volatiles (Friedman, χ^2^_(4)_ = 120.63, Conover post hoc test with Benjamini-Hochberg correction, P < 0.001). Males on the other hand, were significantly less attracted to traps baited with racemic fuscumol (n = 8) than the other two fuscumol treatments and host plant volatiles, but still significantly higher than a control (n = 3) (Friedman χ^2^_(4)_ = 18.38, Conover post hoc test with Benjamini-Hochberg correction, P < 0.001) (Fig. 4A).

During the field bioassays, we also captured 99 beetles (sexes combined) of *Spondylis buprestoides* (L.) (subfamily Spondylidinae). Treatment means were significantly different (Friedman χ^2^_(4)_ = 28.91, P < 0.01), with traps baited with (*S*)-fuscumol catching significantly more beetles (n = 28) than any other lure treatments (i.e. racemic fuscumol caught 24 beetles, a blend of fuscumol and geranylacetone 23, and host plant volatiles 20 beetles, Friedman χ^2^_(4)_ = 28.91, Conover post hoc test with Benjamini-Hochberg correction, P < 0.001). Control traps caught only four *S. buprestoides*, significantly less than any of the lure treatments (Friedman χ^2^_(4)_ = 28.91, Conover post hoc with Benjamini-Hochberg correction, P < 0.001, for all comparisons).

In addition, a few other cerambycid species from the genera *Tetropium* (n = 11), *Monochamus* (n = 4), *Leiopus* (n = 1) and *Prionus* (n = 2) were captured in the pheromone-baited traps, but none in consistent or significant numbers. Furthermore, a click beetle, *Stenagostus rufus* (De Geer) (a rare species in Slovenia, unpub. data) was trapped multiple times across trap transects (overall n = 27), and exclusively females were captured. However, only a blend of fuscumol and geranylacetone (i.e. FG-treatment) was significantly attractive to female *S. rufus* (n = 15 females, Friedman’s, χ^2^_(4)_ = 10.38, Conover post hoc test with Benjamini-Hochberg correction, P < 0.05) (Fig. 4B).

## Discussion

The results of this study expand our knowledge of the pheromone chemistry within the cerambycid subfamily Spondylinae. The identification of the *A. rusticus* pheromone provides the basis for development of effective methods for monitoring and managing this, and possibly other pest and invasive *Arhopalus* species that may vector serious phytopathogens such as the pinewood nematode. The pheromone of *A. rusticus* should provide a sensitive, cost-effective, and environmentally safe tool for early detection and rapid response in order to prevent its establishment and spread in non-native areas. In regions where this species is endemic, data on its abundance and population trends from pheromone-based trapping would provide the information necessary to make informed pest management decisions.

The analytical and bioassay data presented here showed that (*S*)-fuscumol is the major and likely only component of the male-produced aggregation-sex pheromone of *A. rusticus*. The role of geranylacetone, the minor component identified in extracts from *A. rusticus* males, remains uncertain, because the blend of fuscumol with geranylacetone, in the ratio tested was less attractive to females than fuscumol alone, and actually caused attraction of *A. rusticus* females to drop significantly compared to fuscumol alone, suggesting a deterrent effect for females. In further studies aimed at optimizing the attractant lure for practical applications we will also test different ratios of a minor pheromone component, (geranylacetone), on attraction to the major component. Geranylacetone has been demonstrated to be an intermediate in the biosynthesis of fuscumol in two spondylidine species^29^, and it may have some role in mediating heterospecific attraction among co-occurring and closely related *Arhopalus* species, as has been shown for the minor components of pheromone blends of some other cerambycid species^30–33^.

Both sexes of *A. rusticus* were attracted to fuscumol indicating that this compound is an aggregation-sex pheromone^34^, as is the case for all other species in the subfamilies Spondylinae and Lamiinae that use fuscumol or fuscumol acetate as pheromone components^26^. However, the sex ratio of adults attracted to racemic or (*S*)-fuscumol was highly female biased. Whereas this might suggest that *A. rusticus* females are more strongly attracted to fuscumol than males, but, there is little information on the natural sex ratio, and sex ratios in traps may also be influenced by factors such as asynchronous maturation, mating status, different orientation mechanisms, and different distribution patterns between the sexes^22,35^. However, for possible control programs based on pheromones, a lure that strongly attracted females would be more advantageous than a male-biased attractant, because removing females from a population would likely result in larger effects on the subsequent generation than removing males^36^.

Similar numbers of *A. rusticus* were caught in traps baited with either racemic or (*S*)-fuscumol, suggesting that the (*R*)-enantiomer does not inhibit attraction of this species. This is useful for practical purposes because it means that the relatively cheap racemic compound, rather than the much more expensive (*S*)-enantiomer, can be used as an effective lure for detection and management of *A. rusticus*. Because pheromone structures are frequently conserved within related cerambycid taxa, it is also possible, and even likely, that other *Arhopalus* species will be found to produce and respond to fuscumol or closely related analogs. For example, males of the North American congener *Arhopalus productus* LeConte have been shown to produce (*S*)-fuscumol and geranylacetone (JGM, unpub. data). There is also increasing evidence that the fuscumol motif is actually shared more broadly. For example, within the same tribe (Asemini) as *A. rusticus*, *Asemum nitidum* LeConte produces and is attracted to (*S*)-fuscumol and geranylacetone^37^, *Asemum caseyi* Linsley produces and is attracted to geranylacetone^37^, and several *Tetropium* species produce and are attracted to (*S*)-fuscumol^37,38^. Furthermore, during our bioassays, we caught significant numbers of *Spondylis buprestoides*, in a different tribe (Spondylidini), suggesting that this pheromone motif may be conserved throughout the pheromones of the subfamily Spondylidinae.

To date, several studies^23,39–47^ have demonstrated significant attraction of spondylidine species, and specifically *Arhopalus* species, to ethanol (released as a by-product of anaerobic degradation of damaged or dead trees^48^, and/or other host plant volatiles such as α-pinene (a defence compound) produced by conifers against insects and pathogens^49–51^, for review see^26^. We found that a mixture of ethanol and α-pinene was attractive to both sexes of *A. rusticus*, but the host volatiles were not as attractive (at least for females) as the racemic or (*S*)-fuscumol lures. However, plant kairomones may strongly influence the attraction of insects to their sex or aggregation pheromones (e.g.^52–56^). For example, α-pinene was found to strongly synergize attraction of *Tetropium fuscum* to its aggregation-sex pheromone (fuscumol)^43^. Thus, combining host plant volatiles with fuscumol may increase attraction of *A. rusticus*. This will be tested in ongoing trials aimed at optimizing lures and their release rates, traps, and trapping protocols for this species.

The capture of significant numbers of the click beetle *Stenagostus rufus* in traps baited with a blend of fuscumol and geranylacetone suggested that these compounds might be mimicking the sex pheromone of this species. In particular, a number of click beetle sex pheromones have terpenoid motifs^57^, and very recently, fuscumol acetate was shown to be an excellent mimic of methyl dihydrofarnesoate, the sex pheromone of two North American click beetle species in the genus *Cardiophorus*^58^. However if the compound were a sex pheromone we would expect that males would be attracted. Alternatively, predatory click beetles have been shown to exploit the pheromones of heterospecifics as a means of finding their prey. For example, the click beetle *Elater ferrugineus* L. is attracted to the pheromone of its prey, the scarab beetle *Osmoderma eremita* Scopoli^59^. In particular, larvae of *S. rufus*, have often been associated with larvae of longhorn beetles, such as *A. rusticus* and *Rhagium inquisitor*, *Spondylis buprestoides*, and *Strictoleptura rubra*, living together on the same stage of decaying wood, and attacking the cerambycid larvae^18^. This further indicates that the pheromones of cerambycids might function as kairomones for predatory click beetles, mimicking cues or signals associated with hosts, and particularly, oviposition hosts, rather then mimicking sex pheromone of click beetles. *S. rufus* is on the European Red List, (European IUCN Red List) currently listed as “LC” (least concern), but found locally in small populations, and nationally or regionally classified as threatened or near threatened in several European countries, such as Italy, Norway, Sweden, Finland, and others^60–62^. Thus, our study also provides an important first step in investigation of the chemical ecology of this under-recorded and vulnerable insect species.

There are a number of possible practical applications for the *A. rusticus* pheromone, alone or in combination with host plant volatiles. First, the pheromone could be used as a standardized and targeted sampling tool in studies of the biology and life cycle of this nocturnal species. Previous studies have relied on more time- and effort-intensive visual surveys and manual collections of adults from logs randomly selected in the field, or have used generic host plant volatile attractants and light traps to sample *Arhopalus* species (e.g.^22,23,63^). Pheromone-based trapping would also enable long-term and large-scale field surveys that could, in combination with mark-release-recapture experiments, improve our understanding of the dispersal behaviour of *A. rusticus*, provide more reliable estimates of population size, population dynamics, and seasonal phenology in relation to biotic (e.g. predation by click beetles) and abiotic factors (e.g. climate change, storms and fires), and clarify habitat preferences^64–66^.

Second, pheromone-baited traps could provide effective and economically viable tools for surveillance and early detection of *A. rusticus* and possibly other invasive *Arhopalus* species around ports of entry, and particularly around warehouses and other shipping facilities where sealed shipping containers are opened for distribution of their contents. For example, *Arhopalus* was found to be one of the five most frequently intercepted cerambycid genera in six US ports during the inspection of solid wooden packing material^67^. Several recent studies have highlighted the effectiveness of pheromone-baited traps for sampling cerambycids and detecting incursions of exotic species (e.g.,^27,28,68^). Pheromone-baited traps would also facilitate the ongoing monitoring efforts to study the distribution and rate of expansion of *A. rusticus* populations as it continues to spread in new areas of the world which it has invaded^10,14,22^.

Third, fumigation is commonly employed as a phytosanitary treatment for export logs to prevent the spread of target pests into new areas of the world^69^. However, there is heavy pressure to reduce or eliminate the use of fumigants such as methyl bromide, a known ozone destroyer^70,71^. In this context, pheromone-baited traps, along with other environmentally benign measures (e.g. heat treatment, light trapping) may have a role in assessing the need for and efficacy of phytosanitary treatments for quarantine risk management of post-harvest export logs^72^.

Fourth, the *A. rusticus* pheromone could be exploited in management strategies for beetle-vectored phytopathogenic microorganisms such as the pinewood nematode *Bursaphelenchus xylophilus*^45^, which is causing severe ecological and economic damage in conifer forests in areas of Asia and Europe, which it has invaded, and the nematode is under quarantine within the European Union^73–75^.

The growing evidence that fuscumol and its analogues appear to be conserved pheromone structures within both the subfamilies Spondylidinae and Lamiinae (e.g.^27,76–81^) also has major implications for invasion biology. Specifically, it is a prerequisite for reproduction that males and females encounter each other and mate, but the sexes are less likely to locate one another successfully at low densities (i.e. Allee effect^82^). Here, we highlight the concept of the Allee effect in mating success, and the dynamics of biological invasions and consequently in risk assessments of invasive species^26,83^ in the context of *A. rusticus* and related species that use fuscumol or related compounds as their pheromones. The studies cited above have shown that fuscumol is a common pheromone motif among cerambycid species from several continents. When an exotic species using fuscumol-type compounds as its pheromone arrives in a new country, it is possible and even likely that some of the native species will produce the same or very similar pheromone compounds. Through shared pheromones, these endemic species may create a barrier to invasion by disrupting the ability of the invader to find a mate, thus hindering reproduction, population growth, and establishment of the invader^84^.

## Methods and Materials

### Source of insects

During field screening of known cerambycid pheromone components (i.e. racemic 2,3-alkanediols, 2-hydroxy-3-alkanones, 3-hydroxy-2-alkanones, (*E*)-6,10-dimethyl-5,9-undecadien-2-ol (= fuscumol), fuscumol acetate, and 2-(undecyloxy)ethanol (= monochamol, formulated as galloprotect) conducted from July 18^th^ to August 10^th^ in 2013 in the forest of Krim mountain (lat. 45.9611, lon. 14.4600°, 502 m a.s.l.) in Slovenia, several *A. rusticus* beetles were trapped live in the baited black, cross-vane flight-intercept panel traps (1.1 m high × 0.3 m wide, modelled after cross-vane panel traps WitaPrall IntPt–Nassfalle, sold by Witasek PflanzenSchutz GmbH, Austria). The beetles were sexed using the sexually dimorphic characters of antennal length and pronotum length/pronotum width ratio^14^, and then used for collection of headspace volatiles.

### Collection and analysis of beetle volatiles

All beetles used for headspace collections were active and apparently healthy, and were held under ambient conditions (23 ± 1 °C, and 40–50% RH, natural light conditions) for 24 hr before being used for collection of their headspace odours. Volatiles were collected from males (n = 5) and females (n = 5) under ambient laboratory conditions between July 19–26. Individual beetles were placed in modified 250 ml Ball Mason-style canning jars that contained paper tissues (Kimtech Science Precision Wipes, Kimberly-Clark, USA, moistened with distilled water) and a needle-less twig of Norway spruce (*Picea abies* (L.) Karst.) (2 cm long, 0.5 cm wide**)**. The jar lids were fitted with a Teflon liner and two brass bulkhead unions (Swagelok, San Diego Valve and Fitting Co., San Diego CA, USA), for attachment of inlet and outlet air lines. The inlet line was connected to a 2 cm diameter × 20 cm long copper tube filled with activated charcoal granules to clean the incoming air. The outlet line was fitted with a volatiles trap consisting of a glass tube (4 mm diameter and 30 mm long) containing a 10 mm long bed of thermally-desorbed activated charcoal (100–200 mesh; Fisher Scientific, Pittsburgh, PA, USA) held in place by Soxhlet-extracted (ether) glass wool plugs. The collection tube was connected to a flow meter-controlled vacuum source. Charcoal-filtered air was pulled through the chamber and collector at ~200 ml/min. Volatiles were collected for 3 d, after which the traps were extracted with dichloromethane (3 rinses, total volume of ca. 500 μl). Extracts were stored in a refrigerator (2 °C) until used for analyses.

Extracts were analysed at the University of California, Riverside, by coupled gas chromatography-mass spectrometry (GC-MS), in splitless mode using an HP 6890 GC coupled to an HP5973 mass selective detector (Hewlett-Packard, now Agilent, Santa Clara, CA, USA). The GC was fitted with a DB-17 column (30 m × 0.25 mm × 0.25 μm film; J &W Scientific, Folsom CA, USA), and the oven temperature was programmed from 40 °C for 1 min, 10 °C min^−1^ to 280 °C, with helium carrier gas. Compounds were conclusively identified by matching their retention times and mass spectra with those of authentic standards.

To determine the absolute configuration of the male-specific compound fuscumol, an aliquot of an extract was acetylated by adding 100 µl of acetyl chloride solution (1% v/v in dry methylene chloride) and 100 µl of pyridine solution (20 µl + 1–2 mg dimethylaminopyridine in 1 ml dry methylene chloride). The mixture was stirred for 1 hr at room temperature. The excess acetyl chloride was destroyed by addition of ethanol (5 microliters), followed by stirring for an additional 1 hr. Most of the methylene chloride was then removed by blowing down under a stream of nitrogen. The sample was then partitioned between 1 ml of 1 M aqueous HCl and 1 ml hexane, vortexing for 30 sec, followed by removal of the top hexane layer, and washing it with 1 ml of saturated aqueous NaHCO_3_ solution. After drying over anhydrous Na_2_SO_4_ the hexane layer was concentrated under a stream of nitrogen and analyzed by GC on a chiral stationary phase Cyclodex B column (J&W Scientific, 30 m × 0.25 mm id × 0.25 um film) in splitless mode, with the oven temperature programmed from 50 °C for 1 min, then ramped at 3 °C to 220 °C. Authentic standards of (*R*)- and (*S*)-fuscumol acetate were analysed under the same conditions, and as final proof of the configuration of the insect-produced compound, it was coinjected with the (*S*)-fuscumol acetate standard, resulting in a single peak.

### Sources of chemicals

Racemic (*E*)-6,10-dimethyl-5,9-undecadien-2-ol (fuscumol) and fuscumol acetate, and (*E*)-6,10-dimethyl-5,9-undecadien-2-one (geranylacetone) were purchased from Bedoukian Research (Danbury CT, USA). Isopropanol, ethanol, and α-pinene were purchased from Sigma-Aldrich, Steinheim, Germany. (*S*)- and (*R*)-fuscumol were prepared by enzyme-based kinetic resolution of racemic fuscumol as described in Hughes and coworkers^79^.

### Field bioassay of synthetic compounds

The synthetic pheromone candidate and a blend of host volatiles were tested in a field bioassay at Velika ravan in Slovenia (lat. 46°09′, lon. 14° 25′, 350 m a.s.l.), in a forest dominated by conifers including silver fir (*Abies alba* Mill.), Norway spruce (*Picea abies* L. Karst.), and pines (*Pinus* sp.) from 1 July to 2 September, 2014. The mean daily temperature for July and August in Ljubljana was 20.8 °C and 19.6 °C, respectively^85,86^. We used five spatial replicates that were separated by at least 300 m. Each spatial replicate consisted of one of each treatment and a solvent control. The treatments were as follows: 1) (*S*)-fuscumol (25 mg in 1 ml of isopropanol); 2) racemic fuscumol (50 mg in 1 ml of isopropanol); 3) the host plant volatiles ethanol (1 ml) and α-pinene (2 ml); 4) racemic fuscumol + geranylacetone (50 mg racemic fuscumol + 2.5 mg geranylacetone in 1 ml isopropanol); 5) 1 ml of isopropanol as a control. For dispensing pheromone, ethanol, and the solvent control we used clear low-density polyethylene press-seal bags (~5 × 7.5 cm, 51 μm wall thickness, Fisher Scientific, Pittsburgh, PA, USA), whereas α-pinene was dispensed from 50 ml conical-bottomed polypropylene centrifuge tubes (T420-3, external diameter: 29 mm, height: 118 mm, Simport Scientific, Canada, sterile with green caps) with a 2.5 mm hole drilled through in the cap. Estimated release rate of fuscumol in isopropanol from polyethylene press-seal bags, by aeration, was 150 μgrams/day (at 30 °C +/− 1 °C in a climate controlled room) (Halloran and Hanks, unpub. data).

Pheromone solutions were prepared in advance and kept at −20 °C until needed. Lures were loaded with a pipette at the field site immediately before being deployed. Lures were suspended with wire in the central open area of custom-made flight-intercept panel traps as described above, suspended from trees. The traps were painted with Fluon emulsion (Insect-a-Slip Insect Barrier-Fluon, Bioquip Products Inc., Rancho Dominguez, CA, USA) to render trap surfaces slippery. Trap collection cups (white plastic, 8 cm diameter × 17 cm height) were filled with 200 ml of saturated aqueous NaCl solution to preserve captured beetles. For all experiments, traps were placed 20–25 m apart in transects, suspended from tree branches at a height of 1.5–2 m on randomly selected tree species (e.g. *Picea*, *Abies*), and treatments initially assigned randomly to traps. Traps were checked once weekly (total 10 trap checks) and at each check, the traps were rotated one position down the transect, to control for position effects.

### Statistical analyses

Field bioassay replicates were based on temporal (i.e. 10 trap checks) and spatial replications (i.e. five). Differences between mean numbers of beetles, males and females separately, caught per treatment blocked by site and date, were tested using the nonparametric Friedman’s test (because data violated the equal variances assumption of ANOVA^87^) followed by the Conover multiple comparison test^88^, and corrected by the Benjamini-Hochberg procedure^89^. Replicates that contained no specimens were dropped from analyses. The exact binominal test^90^ was used to test whether the sex ratio differed significantly from 0.5. All statistical analyses were conducted with R software, version R 3.5.0 (Copyright (C) 2018 The R Foundation for Statistical Computing Platform^91^).

## Data Availability

Data are the property of the National Institute of Biology (NIB), maintained on government computers, and are of public record. Bioassay data are and the specimens of *A. rusticus* and other species are retained by the authors at NIB and are available by request.
